# The stability investigation of variable viscosity control in the human‐robot interaction

**DOI:** 10.1002/rcs.2416

**Published:** 2022-05-30

**Authors:** Lin Dong, Nicolas Perrin, Florian Richer, Agnes Roby‐Brami, Guillaume Morel

**Affiliations:** ^1^ Institut des Systèmes Intelligents et de Robotique Sorbonne Universités UPMC Univ. Paris 06 Paris France; ^2^ Present address: Center on Frontiers of Computing Studies, Peking University, Beijing, 100089 China.

**Keywords:** co‐manipulation, HRI, human‐robot interaction, impedance control, stability, viscosity control

## Abstract

**Background:**

For many co‐manipulative applications, variable damping is a valuable feature provided by robots. One approach is implementing a high viscosity at low velocities and a low viscosity at high velocities. This, however, is proven to have the possibility to alter human natural motion performance.

**Methods:**

We show that the distortion is caused by the viscosity drop resulting in robot's resistance to motion. To address this, a method for stably achieving the desired behaviour is presented. It involves leveraging a first‐order linear filter to slow the viscosity variation down.

**Results:**

The proposition is supported by a theoretical analysis using a robotic model. Meanwhile, the user performance in human‐robot experiments gets significantly improved, showing the practical efficiency in real applications.

**Conclusions:**

This paper discusses the variable viscosity control in the context of co‐manipulation. An instability problem and its solution were theoretically shown and experimentally evidenced through human‐robot experiments.

## 1 INTRODUCTION

Co‐manipulation is a paradigm in which a robot and a person manipulate an object or a tool at the same time.^1^, ^2^, ^3^ The co‐manipulation combines the advantages of human operators, such as quick learning and adaptability,^4^, ^5^ with the advantages of robots, such as the capacity to improve precision and do repetitive jobs without tiring.^6^ Therefore, nowadays, the concept of co‐manipulation is applied to many tasks so as to optimise ergonomics and to enhance gesture quality.^7^, ^8^, ^9^ For example, in a human‐robot co‐manipulation sawing task,^10^ the authors leverage a myoelectric feedback interface to online estimate human muscle fatigue level and then control the robot to adapt its physical behaviour to the human motor fatigue with the learnt task execution skill. The authors in^11^ proposes a real‐time model‐based reinforcement learning impedance controller to assist human operators in a collaborative lifting task by online optimising the stiffness and damping impedance control parameters to minimise the human effort. The authors in^12^ demonstrate that a collaborative industrial robot with active compliance controller works with a human operator to disassemble the press‐fitted components of an automotive water pump.

Impedance control is a standard paradigm for controlling a co‐manipulator in a co‐manipulative task.^13^, ^14^ While elastic fields (stiffness) are used for guidance through virtual fixtures, viscous fields provide appropriate damping or tremor filtering.^15^ Furthermore, a co‐manipulator may vary its impedance during manipulation activities to give adaptive assistance to changing operating circumstances.

This method was first presented in^16^ for assisting point‐to‐point movements. It is based upon the experimental finding that when a human operator collaborates with another one to transport an object along a linear path, the operator's arm viscosity decreases at a rapid rate. A robot controller is implemented to imitate this behaviour based on the above‐mentioned observation: the viscosity is set large for low velocities while it is set a small value after the velocity reaches the threshold. Consequently, human‐robot co‐manipulative tasks have been demonstrated to follow similar trajectories as in human‐human co‐manipulative tasks. According to the authors in,^17^ this controller is then updated to be “optimal”, that is, the value of viscosity coefficient is an exponential function with respect to time once the threshold is achieved. The resulting velocity profiles of collaborative point‐to‐point motions have the bell shape of minimal jerk trajectories, which, according to the authors, indicates that the movements are “natural”.^18^ Meanwhile, the same experiment conducted using an abrupt change of viscosity, as proposed in,^16^ induces degraded control performance during point‐to‐point movements. This suggests that the way the viscosity variation is dynamically programed impacts the coupled dynamics.

The variable viscosity method is also used for more advanced tasks. In,^19^ an intention‐driven controller is proposed for compliantly‐driven robots and tested on a lower‐limb exoskeleton. The damping coefficient of the controller is online adjusted with the adaptation of the human motion intention through weighting function to suit human behaviour for better collaboration. In a manual welding task with robot assistance,^20^ the damping coefficient is designed to be a piece‐wise linear function of the robot velocity norm. Given the reported performance improvement, as well as the simplicity of the implementation, this controller is used as a starting point of our research.

Our investigation concerns the dynamic behaviour of the couple user + co‐manipulator. In particular, we are interested in the stabilisation of a movement at a given desired velocity. In Section 2.1, we first study this question from a theoretical perspective, within a robotics framework. To this aim, we study the coupling between a conventional velocity controlled robot and the co‐manipulator driven by the controller proposed in.^20^ We show that, under certain conditions, the coupling may exhibit unstable behaviour. In Section 2.2, we propose a solution to stabilise the coupled behaviour. It consists in introducing a secondary linear dynamics to slow down the viscosity coefficient time variations. To complement the theoretical analysis, in Section 3.1, we conduct experiments with human subjects trying to regulate their hand velocity while connected to a co‐manipulator equipped with the variable viscosity controller. We observe oscillatory behaviours when the unstable conditions of the robotics‐based analysis are encountered. Then the human experiments in Section 3.2 (reproducing those of Section 3.1) validate the effectiveness of the proposed method.

## 2 MATERIALS AND METHODS

### 2.1 Variable viscosity coefficient

#### 2.1.1 Basic control law

This paper is part of a research aimed to study co‐manipulation for minimally invasive surgery. Different from tele‐surgery, the surgeon and the co‐manipulator together hold and maneuver the instrument during the operation. We consider a co‐manipulative robot named Achilles^21^ programed to assist a human subject. Achilles is a light‐weight robot fabricated by Haption company, France, see its sketch in Figure 3. It has 6 revolute joints: first three are actuated and last three are passive. The last three joints intersect at a point *P*, thus forming a spherical passive wrist with *P* as the wrist centre.

When the human subject moves his/her hand, the robotic device desired behaviour consists in exhibiting:

- a high viscosity for low hand velocities, in the aim of smoothing fine gestures;
- a low viscosity at high velocities, in the aim of limiting the viscous resisting force during large and low precision movements imposed by the user.

The user holds a handle connected to the end‐effector through the passive spherical wrist centred at point *P*. The device control input is a force $f\in R^{3}$ exerted at point *P*. Its outputs consist in position $x\in R^{3}$ and velocity $v\in R^{3}$ of *P*, obtained from the robot sensors and used as system outputs.

With such a device, we can programme a viscosity controller as:

(1) f=−bv.

A variable viscosity control can be implemented by computing the viscosity coefficient b∈R as a direct function (static map) of the norm of the velocity:

(2) $$b=b_{\max}\cdot \lambda \left(\left\|v\right\|\right),$$

with *b*_max_ the maximal viscosity and 0 ≤ *λ* ≤ 1.

A simple implementation, proposed in,^20^ consists in linearly interpolating the viscosity coefficient between two values, *b*_min_ and *b*_max_, with 0 < *b*_min_ < *b*_max_:

(3) $$\lambda =\left\{\begin{array}{c} 1,\;\text{if}\;\|v\|<v_{\min},\;\\ \frac{b_{\min}}{b_{\max}},\;\text{if}\;\|v\|>v_{\max},\;\\ 1-\frac{\|v\|-v_{\min}}{v_{\max}-v_{\min}}\left(1-\frac{b_{\min}}{b_{\max}}\right),\;\text{otherwise.}\; \end{array}\right.$$

**FIGURE 1:**
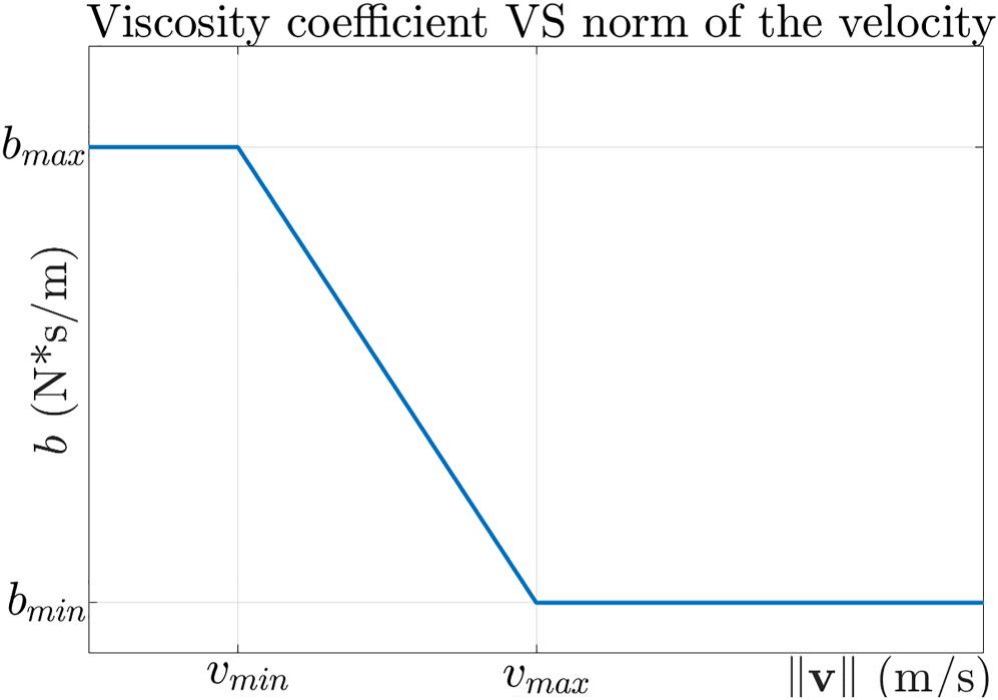
The relation of viscosity coefficient and velocity norm

Figure 1 shows the variation of *b* as a function of ‖**v**‖.

**FIGURE 2:**
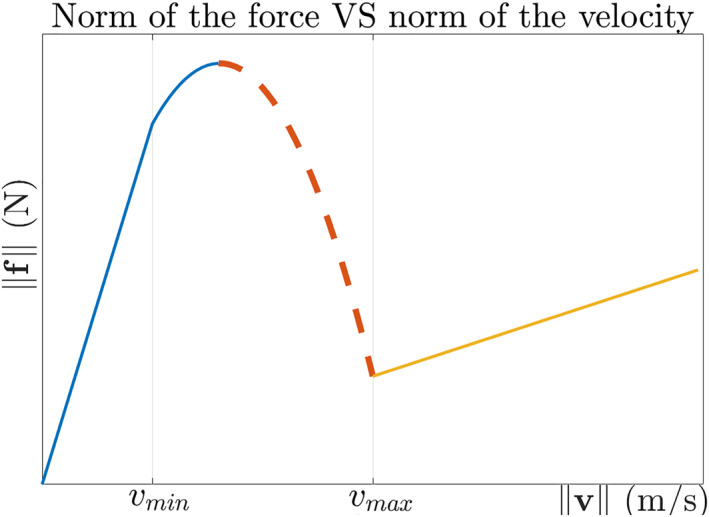
The relation of force norm and velocity norm

This approach has a major issue: within a particular range of velocities, the robot‐exerted resistive force exhibits a decreasing function of the velocity amplitude, as shown in Figure 2. This phenomenon is similar to the Stribeck effect, which is used in tribology to explain the decrease of friction force between the static and dynamic regimes. Taken as a local positive feedback, this behaviour can cause instability at low velocities, leading to stick‐slip movements.^22^ Analogously, we may anticipate the system to have unstable behaviour when the user attempts to maneuver the robot at a velocity that is in the negative slope area (illustrated as dashed red curve in Figure 2).

#### 2.1.2 Theoretical analysis of instability within a robotics framework

**FIGURE 3:**
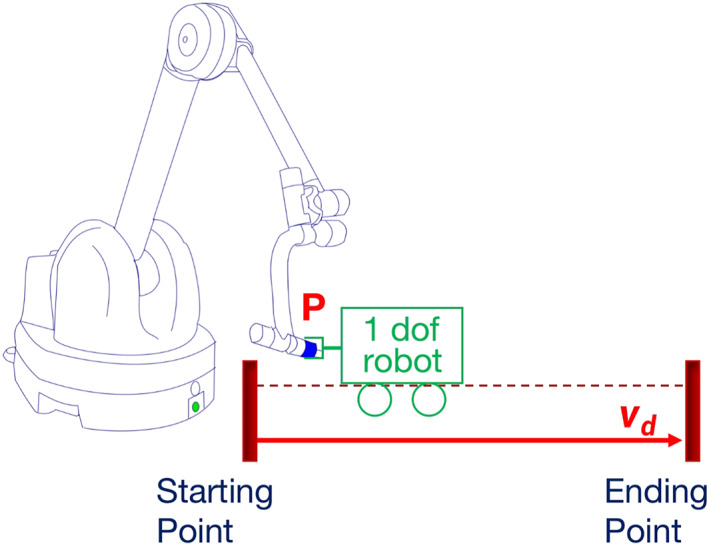
The conceptual illustration of the designed task for instability analysis

We first evaluate the proposed control law from a theoretical perspective. Consider such a task: a 1 dof control robot connects Achilles at its wrist centre point *P* and guides Achilles to move from a given starting point to a given ending point. The movement is supposed to keep a straight line path at constant velocity *v*_*d*_. Achilles, the co‐manipulator, acts as the follower and is controlled according to Equations (1), (2), (3). Figure 3 is an conceptual illustration of the task.

The designed task is a simplified version for theoretical analysis of the practical experiment where a human subject is involved (the experiment detail is provided in Section 3). There are no consensual models available in the literature to precisely depict the complex human dynamic behaviour. Therefore, considering the task requirement of keeping at constant velocity when following a given line, we assume that the assistive control robot exploits a standard PI velocity controller:

(4) $$f_{c}=k_{p}\left(v_{d}-v\right)+k_{i}\int \left(v_{d}-v\right)dt,$$

where *f*_*c*_ is the force exerted by the assistive robot, *k*_*p*_ (resp. *k*_*i*_) is the proportional (resp. integral) velocity controller gain. We model the end‐effector of the assistive robot as a pure mass *m* which is affected by the co‐manipulator force *f* as well as the command force *f*_*c*_, and we write its closed loop behaviour as:

(5) $$m\dot{v}=f+f_{c}=-bv+k_{p}\left(v_{d}-v\right)+k_{i}\int \left(v_{d}-v\right)dt\;.$$

To keep things simple, we'll use the following system state variables:

(6) $$x_{1}=\int \left(v_{d}-v\right)dt,$$

(7) $$x_{2}=v_{d}-v\;.$$

Their derivatives are consequently obtained as:

(8) $$\dot{x_{1}}=x_{2},$$

(9) $$\dot{x_{2}}=-\dot{v}\;.$$

We now limit the study around an equilibrium point, where *b* can be written as linear function of *v*:

(10) $$b=b_{0}-\alpha v\;.$$

*α* is the viscosity slope and *b*_0_ is where the function intersects with the viscosity axis.

According to Equation (7), *v* = *v*_*d*_ − *x*_2_. Then Equation (5) becomes:

(11) $$\dot{v}=\frac{k_{i}}{m}x_{1}+\frac{k_{p}+b_{0}-2\alpha v_{d}}{m}x_{2}+\frac{\alpha }{m}x_{2}^{2}+\frac{\alpha v_{d}^{2}-b_{0}v_{d}}{m}\;.$$

Combining Equations (6), (7), (8), (9), (10), (11), we obtain the system closed‐loop state space equations:

(12) $$\left(\;\begin{array}{c} \dot{x_{1}}\\ \dot{x_{2}} \end{array}\right)=\left(\begin{array}{c} x_{2}\\ -\frac{k_{i}}{m}x_{1}-\frac{k_{p}+b_{0}-2\alpha v_{d}}{m}x_{2}-\frac{\alpha }{m}x_{2}^{2}-\frac{\alpha v_{d}^{2}-b_{0}v_{d}}{m} \end{array}\right).$$

Linearising around the equilibrium point *v* = *v*_*d*_ (corresponding to (*x*_1_, *x*_2_) = (0, 0)), one gets:

(13) $$\left(\begin{array}{c} \dot{x_{1}}\\ \dot{x_{2}} \end{array}\right)=\underset{=:A\left(v_{d}\right)}{\underbrace{\left(\begin{array}{cc} 0 & 1\\ -\frac{k_{i}}{m} & -\frac{k_{p}+b_{0}-2\alpha v_{d}}{m} \end{array}\right)}}\left(\begin{array}{c} x_{1}\\ x_{2} \end{array}\right)\;.$$

The eigenvalues of **A**(*v*_*d*_) are:

(14) $$e_{1,2}=\frac{-T\pm \sqrt{T^{2}-4k_{i}m}}{2m},$$

with *T* = *k*_*p*_ + *b*_0_ − 2*αv*_*d*_. Since *m* > 0, the real parts of *e*_1,2_ are both negative iff

(15) $$\alpha <\frac{b_{0}+k_{p}}{2v_{d}}.$$

We hence draw a conclusion that in a local region the system is asymptotically stable iff $\alpha <\left(b_{0}+k_{p}\right)/\left(2v_{d}\right)$. If $\alpha \geq \left(b_{0}+k_{p}\right)/\left(2v_{d}\right)$, the equilibrium becomes unstable. For the above velocity control task, the stability condition is described as: when a variable viscosity coefficient with slope *α* is given, to keep the velocity at *v*_*d*_, the stiffness *k*_*p*_ must be high enough to ensure the system locally stable.

### 2.2 Our proposition to the instability problem

#### 2.2.1 Slowing down viscosity variation by adding a dynamics

In order to solve this instability problem, we apply to the viscosity coefficient a first‐order low pass filter which is therefore named as viscosity coefficient filter. The purpose of adding this filter is to decelerate the viscosity coefficient variation. This further slows down the viscous force variation, giving the users more time to adjust themselves. Thus the control law writes:

(16) $$f=-b_{f}v,$$

with *b*_*f*_ defined as the filtered viscosity coefficient by equation:

(17) $${\dot{b}}_{f}=\frac{b-b_{f}}{\tau },$$

where *τ* is the filter time constant (unit: second), *b* being obtained from the static velocity‐viscosity map used in the previous section.

#### 2.2.2 Theoretical investigation of stability in a robot‐robot configuration

We use the same model established in Section 2.1.2 for the theoretical investigation. Now the system dynamics becomes:

(18) $$m\dot{v}=-b_{f}v+k_{p}\left(v_{d}-v\right)+k_{i}\int \left(v_{d}-v\right)dt\;.$$

Keeping *x*_1_ and *x*_2_ the same as in Equations (6) and (7), we select *x*_3_ as:

(19) $$x_{3}=b_{d}-b_{f}\;.$$

whose derivative is (recall that *b* = *b*_0_ − *αv*, therefore, *b*_*d*_ ≔ *b*(*v*_*d*_)):

(20) $$\dot{x_{3}}=-\dot{b_{f}}$$

(21) $$\begin{array}{c} =\frac{1}{\tau }\left(b_{f}-b_{0}+\alpha v\right)\\ =\frac{1}{\tau }\left(b_{f}-b_{0}+\alpha \left(v_{d}-x_{2}\right)\right)\\ =-\frac{\alpha }{\tau }x_{2}-\frac{1}{\tau }x_{3}+\frac{\alpha v_{d}+b_{d}-b_{0}}{\tau }\;. \end{array}$$

The closed loop dynamics now writes:

(22) $$m\dot{v}=-\left(b_{d}-x_{3}\right)\left(v_{d}-x_{2}\right)+k_{p}x_{2}+k_{i}x_{1},$$

then the derivative of *x*_2_ is:

(23) $$\dot{x_{2}}=-\dot{v}=-\frac{k_{i}}{m}x_{1}-\frac{k_{p}+b_{d}}{m}x_{2}-\frac{v_{d}}{m}x_{3}+\frac{1}{m}x_{2}x_{3}+\frac{b_{d}v_{d}}{m}\;.$$

Equations (8), (21) and (23) together deduce the new state space equations:

(24) $$\left(\begin{array}{c} \dot{x_{1}}\\ \dot{x_{2}}\\ \dot{x_{3}} \end{array}\right)=\left(\begin{array}{c} x_{2}\\ -\frac{k_{i}}{m}x_{1}-\frac{k_{p}+b_{d}}{m}x_{2}-\frac{v_{d}}{m}x_{3}+\frac{1}{m}x_{2}x_{3}+\frac{b_{d}v_{d}}{m}\\ -\frac{\alpha }{\tau }x_{2}-\frac{1}{\tau }x_{3}+\frac{\alpha v_{d}+b_{d}-b_{0}}{\tau } \end{array}\right)\;.$$

At the equilibrium point, we do the Jacobian linearisation:

(25) $$\left(\begin{array}{c} \dot{x_{1}}\\ \dot{x_{2}}\\ \dot{x_{3}} \end{array}\right)=\underset{=:B\left(v\right)}{\underbrace{\left(\begin{array}{ccc} 0 & 1 & 0\\ -\frac{k_{i}}{m} & -\frac{k_{p}+b_{d}}{m}+\frac{1}{m}x_{3} & -\frac{v_{d}}{m}+\frac{1}{m}x_{2}\\ 0 & -\frac{\alpha }{\tau } & -\frac{1}{\tau } \end{array}\right)}}\left(\begin{array}{c} x_{1}\\ x_{2}\\ x_{3} \end{array}\right)\;.$$

At the reference velocity of the PI control, the viscosity achieves the desired value. To put it another way, when *v* = *v*_*d*_, we have *b*_*f*_ = *b*_*d*_. Thus, the system equilibrium point is (*x*_1_, *x*_2_, *x*_3_) = (0, 0, 0), and around this point Equation (25) rewrites as:

(26) $$\left(\begin{array}{c} \dot{x_{1}}\\ \dot{x_{2}}\\ \dot{x_{3}} \end{array}\right)=\underset{=:B\left(\omega \right)}{\underbrace{\left(\begin{array}{ccc} 0 & 1 & 0\\ -\frac{k_{i}}{m} & -\frac{k_{p}+b_{d}}{m} & -\frac{v_{d}}{m}\\ 0 & -\alpha \omega & -\omega \end{array}\right)}}\left(\begin{array}{c} x_{1}\\ x_{2}\\ x_{3} \end{array}\right)\;.$$

where *ω* = 1/*τ*, denotes the first‐order low pass filter frequency.

We do not write down the eigenvalues of **B**(*ω*) since they are too complex to fit here. Instead, we can prove the existence of a neighbourhood around zero for *ω*, for any given (positive) parameters, which ensures that all the eigenvalues of **B**(*ω*) have negative real parts. We write out the characteristic polynomial of **B**(*ω*):

$$\kappa^{3}+\left(\frac{b_{d}+k_{p}}{m}+\omega \right)\kappa^{2}+\left(\frac{b_{d}+k_{p}-\alpha v_{d}}{m}\omega +\frac{k_{i}}{m}\right)\kappa +\frac{k_{i}}{m}\omega \;.$$

When *ω* = 0, the roots are $\kappa_{1,2}\left(0\right)=-\frac{b_{d}+k_{p}}{2m}\pm \sqrt{{\left(\frac{b_{d}+k_{p}}{2m}\right)}^{2}-\frac{k_{i}}{m}}$, which have negative real parts and *κ*_3_(0) = 0.

Eigenvalues as continuous functions of matrix coefficients, for small enough and positive *ω*, *κ*_1,2_(*ω*) are converted into eigenvalues with negative real parts. *κ*_3_(*ω*), on the other hand, is a real root of the following reformulated characteristic polynomial:

$$\kappa^{3}+a\kappa^{2}+b\kappa +c\;.$$

For small enough and positive *ω*, we can easily verify that *a*, *b* and *c* are all positive, since $\frac{b_{d}+k_{p}}{m}>0$, and $\frac{k_{i}}{m}>0$. If *κ* ≥ 0, the characteristic polynomial is certainly not zero. The third eigenvalue is hence guaranteed to be strictly negative.

Eventually we can draw the conclusion that for small enough and positive *ω*, all the eigenvalues of **B**(*ω*) consist of a negative real part. That is to say, regardless of the controller tuning, as long as *τ* is large enough (*i.e.*, variation of *b*_*f*_ slow enough), the system is locally asymptotically stable.

## 3 EXPERIMENT RESULTS AND DISCUSSION

### 3.1 Practical analysis of instability through human‐comanipulator coupled experiments

**FIGURE 4:**
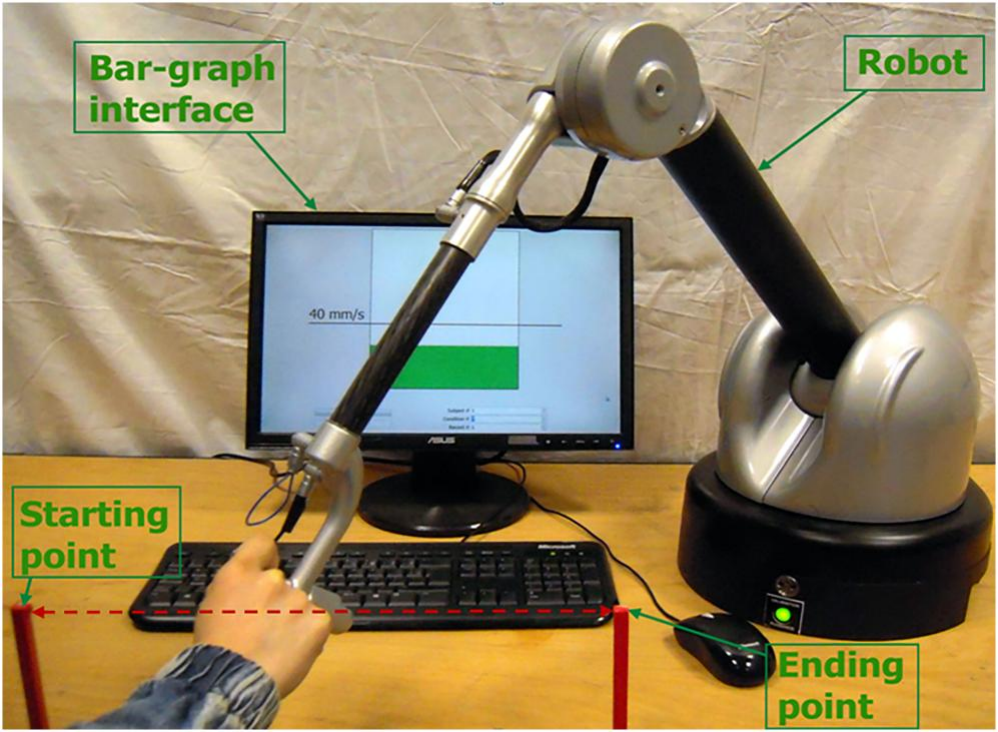
Experimental scene

We conducted experiments to evaluate whether the robot‐robot theoretical instability evidenced in Section 2.1.2 could be observed in a human‐robot configuration. An experimental scene was demonstrated in Figure 4.

Achilles' control input is a pure force at point *P* and therefore it can be easily fitted with the variable viscosity controller given by Equations (1), (2), (3).

For the experimental task a human user replaces the command robot and holds the handle of Achilles. The user is asked to move from the initial point to the final point materialised in the workspace. The distance between the two points is 0.4 m.

**FIGURE 5:**
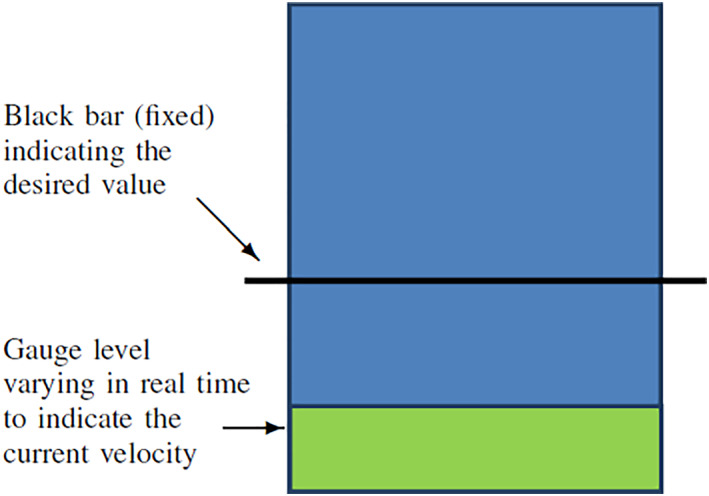
A gauge used to provide the user a visual velocity feedback

The user is asked to move the robot following a straight line between the starting and ending points while at the same time concentrating on the velocity. This is assisted by an interface illustrated in Figure 5, where a gauge provides the user a visual feedback of the current velocity norm. Concretely, the user is asked to keep the velocity at a constant desired value *v*_*d*_ during the movement, indicated by the black bold line in the graph.

**FIGURE 6:**
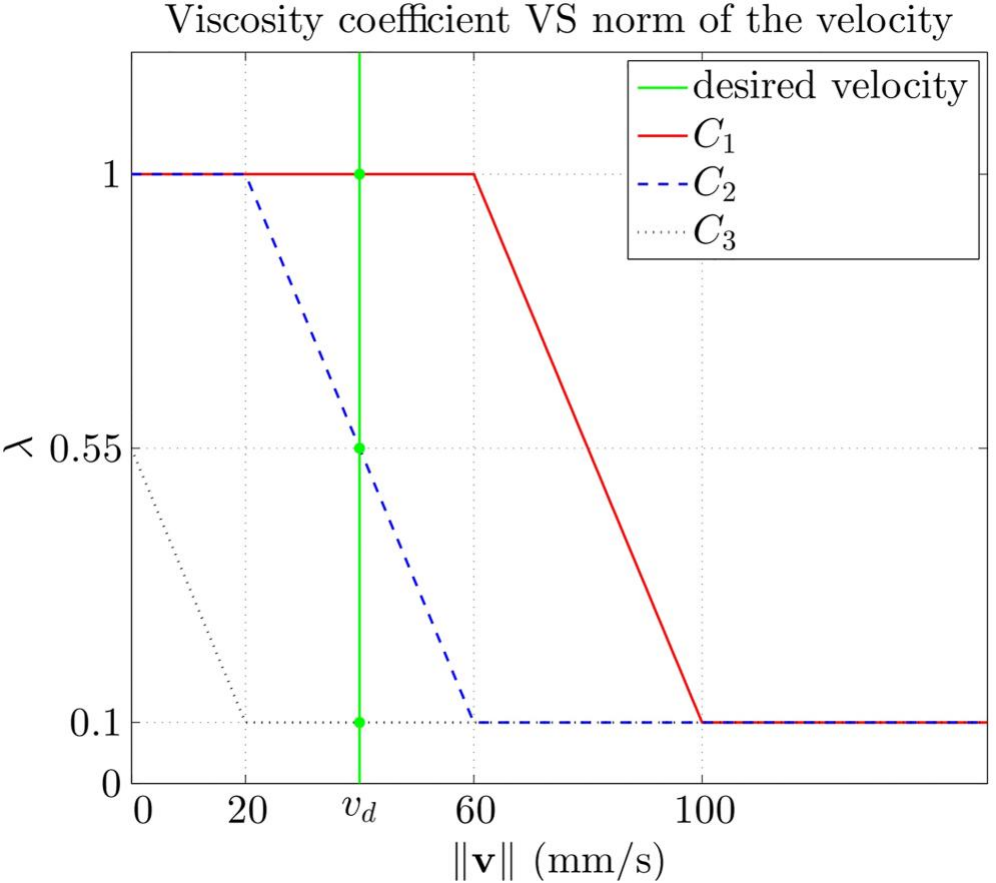
Three designs of *λ*(‖**v**‖). The three experiments share the same desired velocity *v*_*d*_ and the dropping slope

As depicted in Figure 6, three velocity‐to‐viscosity static maps $\lambda \left(\left\|v\right\|\right)$ are used to obtain three different experimental conditions, with a same value of *v*_*d*_.

For Condition *C*_1_, $\lambda \left(\left\|v\right\|\right)$ is specially devised to ensure *v*_*d*_ falls inside the high viscosity coefficient area. For *C*_2_, $\lambda \left(\left\|v\right\|\right)$ is devised to ensure *v*_*d*_ falls inside the viscosity coefficient drop area. For *C*_3_, $\lambda \left(\left\|v\right\|\right)$ is devised to ensure *v*_*d*_ falls inside the low viscosity coefficient area. Notice that, according to the theoretical analysis, if a PI controlled robot was connected to the comanipulator, then stability would be obtained for both *C*_1_ and *C*_3_ (since *α* = 0) while, depending on the control parameters, instability could be observed for *C*_2_.

Ten naive subjects, all right‐handed, participated in the experiment. Conditions are randomly loaded. Before the formal recording, subjects were suggested to take a few trials for every condition so as to get familiar with the required force level for moving Achilles at *v*_*d*_.

**FIGURE 7:**
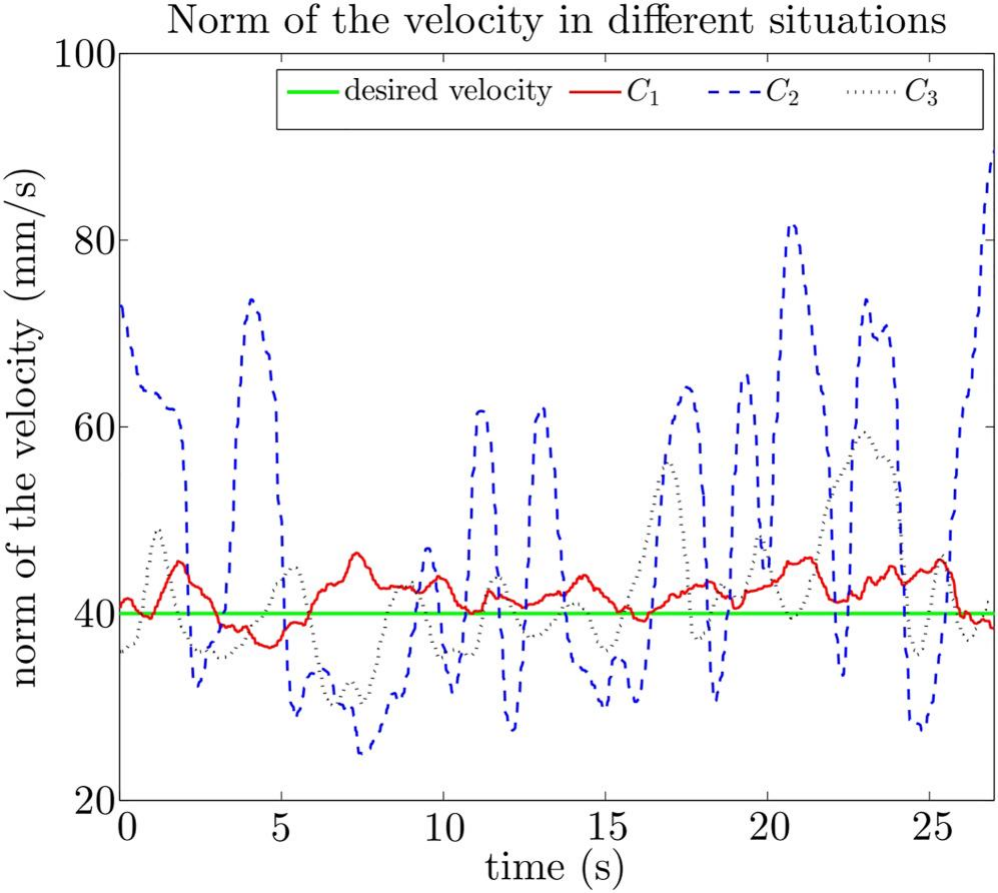
The velocity performance of a random subject under Conditions *C*_1_ − *C*_3_

Figure 7 shows the typical behaviour of a random subject. From the figure, we see that the subject is able to maintain the velocity stable under *C*_1_ and *C*_3_. Further, the high viscosity coefficient under *C*_1_ provides higher damping, leading to less error. The more important phenomenon is the instability observed under *C*_2_: To reach *v*_*d*_, the subject accelerates when initially at low velocity. The velocity acceleration corresponds to the viscosity drop, leading to a decreasing resistance to robot motion. The acceleration is easily getting higher than the subject's expectation, landing to the high velocity region (with *b* = *b*_min_). The subject then adjusts the velocity to slow down, but again than expected, too much deceleration lands to the low velocity region. The final result is that the subject fails to stabilise the velocity at *v*_*d*_. The oscillation between two limit regions resembles the stick‐slip motions observed in.^22^

**FIGURE 8:**
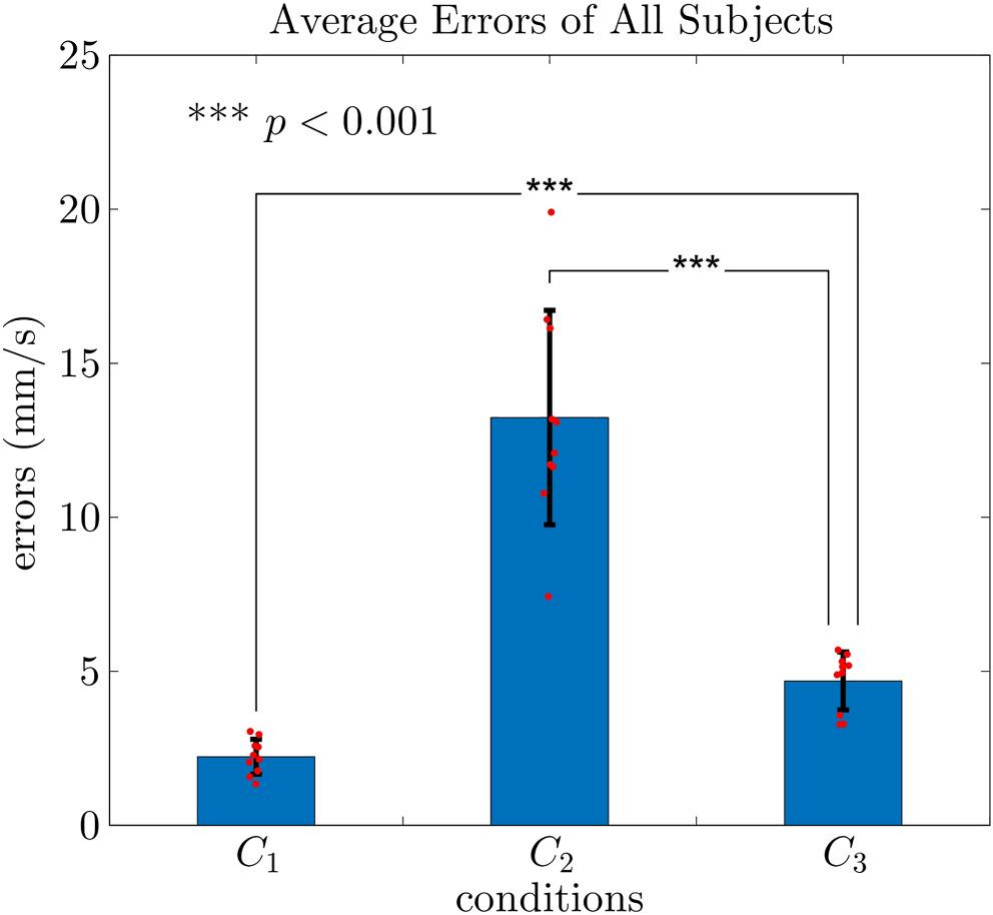
Average errors of all ten subjects under *C*_1_ − *C*_3_

This observation is typical for all subjects. Figure 8 is the Root‐mean‐square (RMS) of velocity errors and the corresponding standard deviations under the 3 conditions. To be more informative, the error of each subject is depicted with a small red dot.

We also conducted a Student's *t*‐test for a quantitative assessment of the performance under *C*_1_ − *C*_3_. The RMS error under *C*_3_ (*μ* = 4.7, *σ* = 0.94, unit: mm/s, hereinafter the same) is more than twice compared with that under *C*_1_ (*μ* = 2.2, *σ* = 0.57), with a statistically significant difference (*p* = 2.7 × 10^−6^ ≪ 0.05). On the contrary, the RMS error under *C*_2_ (*μ* = 13, *σ* = 3.5) is almost three times of that under *C*_3_, and the *p*‐value (*p* = 4.0 × 10^−5^) reveals that the difference is statistically significant.

We hence claim that the system benefits from the viscosity in terms of stability but the dropping viscosity induces the instability. The experimental results are comparable to the theoretical results obtained with a robot‐robot configuration. In the region having a large dropping viscosity, the system is prone to be unstable.

### 3.2 Experimental evaluation within in a human‐robot context

Since the model for the theoretical analysis does not represent a human dynamics (whose model is not precisely known), the stability condition is not appropriate for the practical controller tuning. However, in the current experiment, we have assumed the viscosity parameters in Section 3.1 unchanged. Besides, we add a first‐order low pass filter with *τ* = 1*s* or 2*s*.

The experiment protocol keeps the same as in Section 3.1. Specifically, we add four new conditions. *C*_4_ − *C*_6_ corresponds to *C*_1_ − *C*_3_, but with an additional viscosity filter (*τ* = 1*s*). *C*_7_ corresponds to *C*_2_, with *τ* = 2*s*. The conditions are listed in Table 1.

**TABLE 1:** The seven experimental conditions

Conditions	High viscosity coefficient (stable area)	Medium viscosity coefficient (unstable area)	Low viscosity coefficient (stable area)
No filter τ=0s	$C_{1}$	$C_{2}$	$C_{3}$
Filter τ=1s	$C_{4}$	$C_{5}$	$C_{6}$
Filter τ=2s	NA	$C_{7}$	NA

**FIGURE 9:**
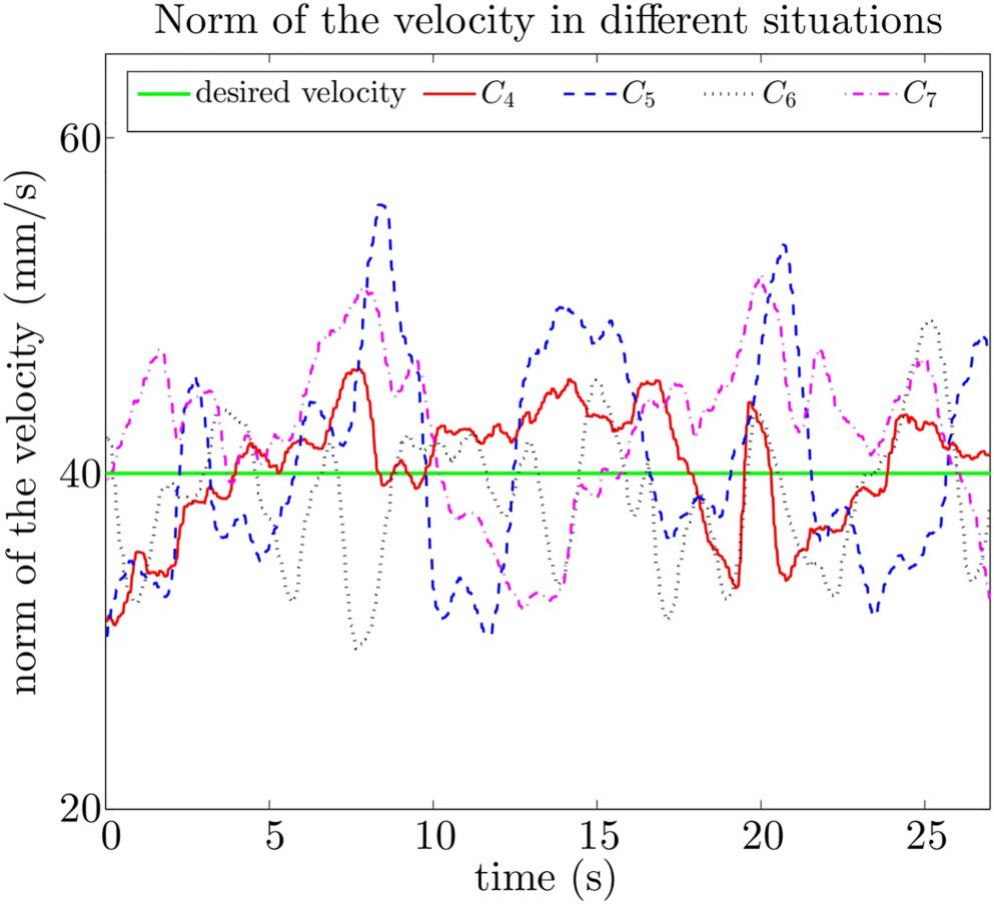
The velocity performance of a random subject under Conditions *C*_4_ − *C*_7_

Figure 9 displays the velocity norm w.r.t time under *C*_4_ − *C*_7_ for the same subject in Figure 7. Under *C*_4_ and *C*_6_, the subject has similar performance as under *C*_1_ and *C*_3_. Under *C*_5_ and *C*_7_, the subject now has the ability to stabilise the velocity at *v*_*d*_, compared with the unstable performance under *C*_2_.

**FIGURE 10:**
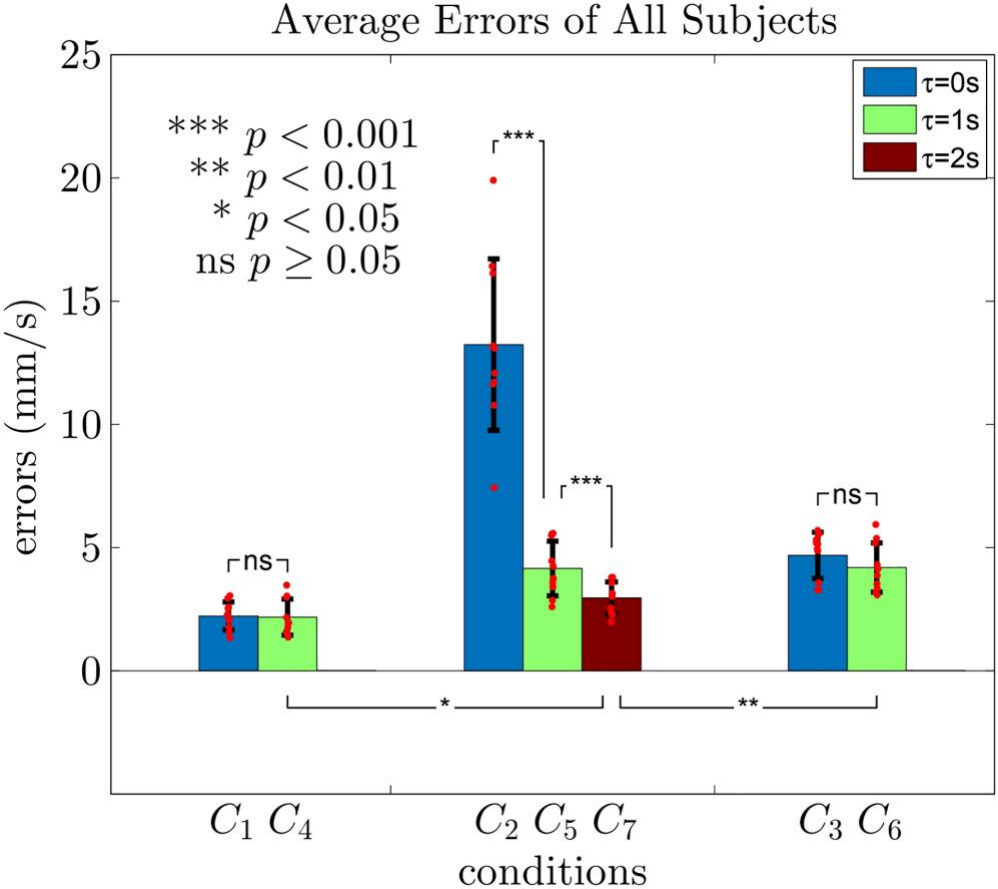
The average errors of all 10 subjects under *C*_1_ − *C*_7_

This observed behaviour is typical for all 10 subjects. Figure 10 is the RMS of velocity errors and the corresponding standard deviation under *C*_1_ − *C*_7_ (results under *C*_1_ − *C*_3_ reproduced from Figure 8). Conditions are organised based on their viscosity regions for better comparison.

The Student's *t*‐test is also conducted. The RMS error under *C*_1_ (*μ* = 2.2, *σ* = 0.57, unit: mm/s, hereinafter the same) is close to that under *C*_4_ (*μ* = 2.2, *σ* = 0.73), evidenced by the statistically insignificant difference (*p* = 0.86). Similarly, the RMS error under *C*_3_ (*μ* = 4.7, *σ* = 0.94) is close to that under *C*_6_ (*μ* = 4.2, *σ* = 1.0) with *p* = 0.25. Therefore, we conclude that an additional viscosity coefficient filter does not degenerate the performance of the original stable areas, which was expected as the viscosity is supposed to be constant (*α* = 0).

As to the RMS errors under *C*_2_ with *τ* = 0*s* (*μ* = 13, *σ* = 3.5), under *C*_5_ with *τ* = 1*s* (*μ* = 4.2, *σ* = 1.1) and under *C*_7_ with *τ* = 2*s* (*μ* = 3.0, *σ* = 0.65), due to the added filter, the values become drastically smaller, evidenced by statistically significant differences of *p*‐values (9.5 × 10^−6^ for *C*_2_ V.S. *C*_5_ and 5.2 × 10^−4^ for *C*_5_ V.S. *C*_7_).

These observations are comparable to what was theoretically established for a robot‐robot configuration: adding a filter with *τ* large enough leads to a locally asymptotically stable coupled system.

## 4 CONCLUSION

This paper discusses about the variable viscosity control in the context of co‐manipulation. The viscosity coefficient decreases as a function of velocity. An instability problem which has not been reported in literature, was theoretically shown using an robotics model and experimentally evidenced through human‐robot experiments. We propose to add a secondary dynamics to slow down the viscosity variation. This proposition is supported by a theoretical analysis within the robotics context, while human‐robot experiments show its practical efficiency in real applications.

We study the instability problem of the variable viscosity control in the context of co‐manipulation. Unlike tele‐manipulated surgical robotic systems such as the da Vinci robot where the slave robotic arms mimic exactly the surgeon's motion, in co‐manipulated robotic surgery, the involvement of human user makes the whole system dynamics different from the dynamics of the master/slave robotic system. For this reason, in the future work, more commercial co‐operative robots with different mechanical structure such as Franka Emika Panda, Kuka iiwa, etc., shall replace Achilles as the co‐manipulator to provide more solid experimental validation. In addition, we shall design more complicated human robot co‐manipulation tasks such as following a given trajectory, virtual fixture guidance,^23^ etc. to further verify the controller stability in the co‐manipulated surgery.

## CONFLICT OF INTEREST

The authors state explicitly that there are no conflict of interest in connection with this article.

## Data Availability

The data that support the findings of this study are available from the corresponding author upon reasonable request.
